# Neutrophils: Their Role in Innate and Adaptive Immunity

**DOI:** 10.1155/2016/1469780

**Published:** 2016-02-24

**Authors:** Carlos Rosales, Nicolas Demaurex, Clifford A. Lowell, Eileen Uribe-Querol

**Affiliations:** ^1^Instituto de Investigaciones Biomédicas, Universidad Nacional Autónoma de México, 04510 Ciudad de México, DF, Mexico; ^2^University of Geneva, 1211 Geneva, Switzerland; ^3^University of California, San Francisco, CA 94143, USA; ^4^Facultad de Odontología, Universidad Nacional Autónoma de México, 04510 Ciudad de México, DF, Mexico

Polymorphonuclear neutrophils (PMNs) are the most abundant leukocytes in the blood and constitute the first line of host defense against numerous infectious pathogens, including bacteria, fungi, and protozoa. Neutrophils are the first leukocytes to migrate from the blood to injured or infected sites for killing pathogens and removing cellular debris. Neutrophils migrate to sites of inflammation and infection where they recognize and phagocytose invading microorganisms, in order to kill them via different cytotoxic mechanisms. This process involves molecular mechanisms that coordinate cell polarization, delivery of receptors, and activation of integrins at the leading edge of neutrophils migrating toward chemoattractants. Once at sites of infection, neutrophils actively phagocytose microorganisms or form neutrophil extracellular traps (NETs) to trap and kill pathogens. Association of the nicotinamide adenine dinucleotide phosphate (NADPH) reduced oxidase complex at the phagosomal membrane for the production of reactive oxygen species (ROS) and delivery of proteolytic enzymes into the phagosome initiate pathogen killing and removal.

In recent years, it has become evident that neutrophils not only have a fundamental role in the acute phase of inflammation when they actively eliminate pathogens, but also are capable of modifying the overall immune response. Neutrophils can do this by exchanging information with macrophages, dendritic cells, and other cells of the adaptive immune system through either soluble mediators or direct cell-cell contact.

To illuminate the complex role of neutrophils in infection, inflammation, and immunity, this special issue has gathered original and review articles that will help us expand our knowledge on neutrophil biology.

As stated before, any neutrophil response begins with migration of these leukocytes to the site of infection or inflammation. Chemotaxis, the directional movement of the cell guided by extracellular chemoattractant gradients, plays an essential role in the recruitment of neutrophils to sites of inflammation. Chemotaxis is mediated by G protein-coupled receptor (GPCR) signaling pathway. The article by X. Xu and T. Jin describes the novel functions of the PLC/PKC/PKD signaling axis in GPCR-mediated chemotaxis of neutrophils. Similarly, the review by J. Gamara et al. explains how the small monomeric GTPases of the Arf family and their guanine exchange factors (GEFs) and GTPase activating proteins (GAPs) participate in GPCR signaling cascades regulating several neutrophil functional responses. The various cell responses to different chemoattractants are highlighted by the studies of R. Vaivoda et al. and M. A. Hidalgo et al. R. Vaivoda et al. describe how the deficiency of CYP4F18, an enzyme that catalyzes hydroxylation of leukotriene B4 (LTB4), does not alter the chemotactic response to LTB4 but causes a twofold increase response to complement component C5a. M. A. Hidalgo et al. describe that although N-formyl-methionyl-leucyl-phenylalanine (fMLF) and Platelet Activating Factor (PAF) induce similar intracellular signaling profiles, only fMLF induces interleukin-8 (IL-8) release and NADPH oxidase activity in neutrophils.

Several microbicidal functions of neutrophils involve the activation of the NADPH oxidase complex for production of reactive oxygen species (ROS) to mediate pathogen killing.

The interesting review by R. C. Allen describes the principles of particle physics and quantum mechanics to develop a fundamental explanation of neutrophil microbicidal metabolism based on ROS atomic properties. Myeloperoxidase, the most abundant neutrophil-granule protein, is known to have potent microbicidal properties. But, recently it has become apparent that myeloperoxidase also participates in regulating adaptive immune responses. The review by D. Odobasic et al. presents an overview on how this enzyme has key roles in various functions of neutrophils in innate and adaptive immunity. When neutrophils cannot kill microorganisms by the classical phagocytosis or degranulation mechanisms, they can also form neutrophil extracellular traps (NETs) to kill microbes. NETs are fibers composed of chromatin and neutrophil-granule proteins and are induced by several pathogens and also some pharmacological stimuli. Antigen-antibody complexes can also induce NET formation. The paper by O. R. Alemán et al. explores direct stimulation of individual Fc*γ* receptors to induce NET formation and finds that only Fc*γ*RIIIb cross-linking induced NET formation in a NADPH oxidase-, PKC-, and ERK-dependent fashion. An intriguing observation on the microbicidal function of neutrophils of older women is reported by J. Bartholomeu-Neto et al. They find that phagocytic and oxidative activities of neutrophils of healthy older women that exercise regularly are higher than those of sedentary older women. The physical condition of each individual was a significant predictor of phagocytosis potential. Clearly, regular exercise contributes to a better innate immune system. However, no mechanism for this beneficial effect is known. This should be an interesting line of future neutrophil research.

Neutrophils are potent regulators of inflammation via the release of proinflammatory factors and several cytokines. The paper by M. R. Tardif et al. describes an alternative secretion pathway in neutrophils for the release of the S100A8/A9 (calprotectin) and S100A12, proinflammatory mediators. The secretion of these cytoplasmic proteins was dependent on the production of ROS and required K^+^ exchanges through ATP-sensitive K^+^ channels. Because neutrophils can also cause tissue damage, their downregulation at the inflammatory site is required for proper resolution of inflammation. The paper by M. A. Sugimoto et al. describes how Annexin A1, an endogenous glucocorticoid-regulated protein, inhibits neutrophil tissue accumulation by reducing leukocyte infiltration and activating neutrophil apoptosis. Annexin A1 also induced macrophage reprogramming toward a resolving phenotype, resulting in reduced production of proinflammatory cytokines and increased release of immunosuppressive and proresolving molecules. Similarly, the paper by M. Cohen-Mazor et al. describes how heparin binds to activated neutrophils and induces apoptosis. These results provide an explanation for the long-known anti-inflammatory effects of heparin.

Neutrophils seem to have dual roles in promoting and controlling inflammation. The mechanisms that control the final outcome are not completely described, but these opposite functions must be tightly balanced. During sepsis, neutrophils are responsible for both the release of cytokines and the phagocytosis of pathogens. But, in SIRS (systemic inflammatory response syndrome), neutrophils contribute to maintaining of a whole-body inflammatory state. In the review by H. Fang et al., the role of neutrophils in these two clinical conditions is described and the therapeutic effect of G-CSF in sepsis is discussed in relation to its function of regulating neutrophil blood levels. Moreover, because neutrophil-derived products can regulate the action of other immune cells and can contribute to the development and chronicity of inflammatory diseases, I. Naegelen et al. propose in their article an original strategy based on linear fitting, to analyze the link between cytokine release and degranulation time. This method could find correlations between granule proteins and cytokines secreted to the inflammatory site. The idea is to be able in the future to predict the type of inflammatory response that neutrophils could induce under certain conditions. In addition, C. F. M. Morris et al. describe in their review how two apparent opposite models of inflammation may be compatible in the outcome of inflammation. The two-hit model states that a first injury (i.e., hit) can serve as a priming event which sequential insults can build on, culminating in a disproportioned inflammatory response to injury. On the other hand, the ischemic preconditioning (IPC) model states that a mild ischemic event, either remote or local, can be protective and can actually attenuate the inflammatory response to the following insults. This article tries to reconcile both models and presents evidence that each of them brings its own unique perspective onto the biology of inflammation. Finally, the dual role of neutrophil function during inflammation is emphasized again in the review by E. Uribe-Querol et al., where the protumor or antitumor character of the tumor-associated neutrophils (TANs) is revised. Recent findings on the mechanisms for neutrophil recruitment to the tumor, for neutrophils supporting tumor progression, and for neutrophil activation to enhance their antitumor functions are presented.

These articles together represent the fascinating flexible functions of neutrophils not only in fighting infections, but also in shaping the immune response and the consequences this may have to important health issues such as resolution of inflammation, autoimmunity, and cancer.

*Carlos Rosales* *Carlos Rosales* *Nicolas Demaurex* *Nicolas Demaurex* *Clifford A. Lowell* *Clifford A. Lowell* *Eileen Uribe-Querol* *Eileen Uribe-Querol*

